# Scoping review of patient-centered care approaches in healthcare

**DOI:** 10.1186/1472-6963-14-271

**Published:** 2014-06-19

**Authors:** Marissa K Constand, Joy C MacDermid, Vanina Dal Bello-Haas, Mary Law

**Affiliations:** 1 School of Rehabilitation Science, McMaster University, 1400 Main Street West, L8S 1C7, Hamilton, Ontario, Canada

**Keywords:** Patient-centered care, Model, Framework

## Abstract

**Background:**

The purpose of this scoping review was to describe how three tenants of patient-centered care provision: communication, partnership, and health promotion are addressed in patient-centered care models/frameworks across the literature.

**Methods:**

A scoping review of literature published in English since 1990 was conducted using Medline, CINAHL, and EMBASE. A key term search strategy was employed using “patient-centered care”, “client-centered care”, “framework” and “model” to identify relevant studies.

**Results:**

Application of the search strategy resulted in a hit total of 101 articles. Nineteen articles met inclusion criteria, of which 12 were review articles; 5 were qualitative research papers; one was a randomized control trial; and one was a prospective study. From these articles, 25 different patient-centered care frameworks/models were identified.

**Conclusions:**

The fact that all identified approaches to patient-centered care incorporated strategies to achieve effective communication, partnership, and health promotion indicates that clinicians can select a patient-centered approach from the literature that best suits their patient’s needs, and be confident that it will satisfy the three core elements of patient-centered care provision. While empirical literature on specific patient-centric frameworks and models was limited, much empiric evidence was sourced for the most consistently defined component of patient-centered care, communication.

## Background

Patient-centered care in healthcare is defined as care provision that is consistent with the values, needs, and desires of patients and is achieved when clinicians involve patients in healthcare discussions and decisions [1]. The Patient Centered Clinical Method identifies that patient-centeredness is achieved in part by understanding patients experiences with illness and disease as well as understanding patients holistically [2]. Patient-centered care is thought to have many benefits and has been proposed as a means of achieving better health outcomes, greater patient satisfaction, and reduced health costs [2]. For example, Cooper and colleagues [3] have identified that in a population of patients receiving physiotherapy for the treatment of chronic low back pain, the provision of patient-centered care helped the physiotherapists to “better understand and manage” their patient’s needs. Furthermore, Cott [4] identified that an improved understanding of patient needs stems from clinicians acknowledging patient perspectives on recovery.

In a multi-site study conducted in primary care physician’s offices servicing members of both urban and rural communities, Little et al. [5] surveyed patient preferences for patient-centered care and suggested that the three main objectives of patient-centered care provision should include effective communication, partnership, and health promotion. Effective communication has been defined as the exploration of the patient’s disease and illness to develop an understanding of the patient’s healthcare experiences [1,2]. Developing a partnership with patients occurs when clinicians and patients find common ground upon which a healthcare plan can be developed mutually [1,2]. Finally, effective health promotion, defined in this study as tailoring healthcare plans based on reflections on the patient’s past health history and current health context, helps ensure that healthcare plans are developed from an understanding of previous healthcare experiences. This approach reduces the risk of failed treatments and ensures optimal use of resources [1,2]. While these three components of patient-centered care have been identified as the elements that are most valued by patients receiving medical attention [5], the extent to which different patient-centered care frameworks and models embrace these three components as core elements, and their application across different disciplines has not been studied. Although rehabilitation is an area of practice where patient-centered care is seen as “the way forward” [6], even here a consistent conceptual framework or model of patient-centered care has yet to be accepted. Clarity on definitions, frameworks, and essential ingredients of patient-centered care is a prerequisite for developing rigorous empirical evidence evaluating patient-centred care and for insuring fidelity when it is implemented. A scoping review approach provides a methodology for determining the state of the evidence on a topic that is especially useful where issues require clarification before rigorous empirical studies are conducted. Therefore, the purpose of this study is to use Arskey and O’Malley’s scoping review methodology to determine the following with respect to patient-centered care frameworks and models:

1. What is the extent and nature of published scientific literature on patient-centered care frameworks and models including the research designs used, areas of clinical practice, and conceptualization of patient-centered care?

2. To what extent do the frameworks and models address the three core components of patient-centered care: effective communication, partnership, and health promotion?

A secondary purpose was to reflect on the depth of evidence surrounding a key component of patient-centered care, effective communication, by charting the published systematic reviews on effective communication practices. This review was conducted as a secondary review in order to identify evidence supporting patient-centered communication that may not be associated with a patient-centered framework or model since effective communication is the most definable and consistent component of patient-centered care.

## Methods

### Identifying relevant studies

Literature published in English between 1990 and 2012 was collected from three databases: Medline, CINAHL, and EMBASE. A key term search strategy was employed using the words “patient-centered care”, “client-centered care”, “framework” and “model”. The terms “framework” and “model” were selected to classify the approaches to patient-centered care provision because they provide standardized methods that can be easily followed and reproduced. A similar search was conducted for systematic reviews that included communication as a title word to identify the most easily accessible systematic reviews addressing communication.

### Study selection

Articles were eligible for inclusion in this review if they described a patient-centered care framework or model being applied to an adult population receiving healthcare. Only articles published since 1990 and written in English were eligible for inclusion in this review. Articles were excluded if they did not pertain to a patient-centered care framework or model, or if did not address a healthcare context. Titles and abstracts of articles were independently reviewed by two authors (MKC and JCM). If articles were representative of the inclusion criteria, the articles went through two full-text independent reviews by two authors (MKC and JCM). If disagreements arose, a third party reviewer would be consulted. A second search was conducted using communication as a keyword, and limiting the retrieval to systematic reviews using Clinical Queries in Medline, and the term systematic review in other databases. Articles were included from the secondary review of the literature if they were systematic reviews identifying effective communication strategies in any healthcare discipline. Studies were excluded if they did not identify communication strategies between clinicians and patients or families.

### Charting data

If an article was eligible for inclusion in this study, data related to the patient-centered care framework or model presented in the article was extracted by the lead author and reviewed by a second author (JCM). Data extracted from the reviewed patient-centered care frameworks and models was entered into data extraction records and synthesized in summary format. Data were systematically charted using the data charting form developed in Microsoft Excel. Information on authorship, article type, population, and patient-centered care approach were recorded on this form. A second data charting form was developed to chart data on the communication systematic reviews identified. Information on clinical context, patient-centered care focus, number of studies reviewed and key findings were recorded on this form.

### Collating, summarising and reporting results

Information that was organized on the data charting forms was employed to collate and report the articles’ approaches towards achieving effective communication, partnership, and health promotion.

## Results

**Figure 1:**
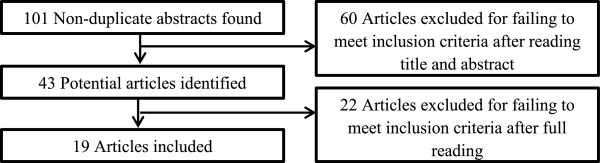
Scoping review process.

From an original hit total of 101 articles, 60 articles were excluded after reading the article title, and 22 articles were excluded after they were read fully (Figure 1). Nineteen articles were selected for inclusion in this review. Twelve of these articles were narrative review articles. The remaining studies included four qualitative research papers, one randomized control trial, and one prospective study. Of the 19 included articles, 25 unique patient-centered care frameworks or models were identified (Table 1). The secondary review conducted on communication strategies yielded a hit total of 69 systematic review articles, 25 of which met inclusion criteria (Table 2).

**Table 1:** Scoping review included articles

**Author**	**Article type**	**Population**	**Patient-centered care approach identified**
Ballweg [7]	Review article	Neonatal Intensive Care Unit	Developmentally Supportive, Family-Centered Care Model
Berger [8]	Review article	Psychiatry	The Tidal Model
Bickler [9]	Review article	Surgery	Patient-Focused Care Model
Boltz [10]	Review article	Geriatrics	Nurses Improving Care for Health System Elders
Booth & MacBride [11]	Review article	Generic	Patient-Centered Clinical Method
Briggs [12]	Review article	Palliative Care/	National Consensus Project for Quality Palliative Care
		Physical Therapy/	
		End of Life Care	Hypothesis Oriented Algorithm for Clinicians
			Framework for Rehabilitation of Neurodegenerative Diseases Framework for Assessment in Oncology Rehabilitation
			Models of Practice in Palliative Care
Browne et al. [13]	Review article	Nursing	Decentralization
Cox [14]	Review article	Psychiatry	Biopsychosocial Model
deLusignan et al. [15]	Review article	Nursing	Model for Patient-Centered Consultations with Nurses in Primary Care
DiGoia et al. [16]	Prospective study	Orthopedics	Patient and Family Centered Collaborative Care
Enguidanos et al. [17]	Randomized control trial	Geriatrics/Psychiatry	Integrated Depression Care Management Model
Ford et al. [18]	Review article	Nursing	RNAO Best Practice Guideline on Client Centered Care
Hantho et al. [19]	Review article	General	Malterud’s Key Questions
			Stuart’s BATHE Model
			The Communication Model
Hatzichristou & Tsimtsiou [20]	Review article	Urology	Patient Centered Model for the Management of Sexual Dysfunction
Kelleher [21]	Review article	Intensive Care	The Synergy Model
Kibicho & Owczarzak [22]	Qualitative research	Pharmacy	Patient-Centered Pharmacy Services
McCormack [23]	Qualitative research	Geriatrics	Authentic Consciousness
Rosvik et al. [24]	Qualitative research	Geriatrics	VIPS Practice Model
van der Eijk et al. [25]	Qualitative research	Neurology	Theoretical Model of Patient Centeredness for Parkinson’s Disease

**Table 2:** Systematic reviews on communication in healthcare

**Systematic review**	**Clinical context**	**PCC focus**	# **Studies reviewed**	**Key findings**
Beck, Daughtridge & Sloane [29]	Primary Care	Yes	22	• Physician behavior linked with positive patient outcomes, adherence, and patient satisfaction
Chan et al. [30]	Pre-operative Care	Yes	11	• Sharing information, family involvement, autonomy, and professionalism are key
				• Knowledgeable clinicians with positive attitudes enhance patient “journey”
Davis et al. [31]	Oncology	Yes	21	• Complementary and alternative medicine use in patients with cancer must be discussed using effective communication skills in order to avoid patients failing to disclose use with clinician
Edwards et al. [32]	Genetics	Yes	28	• Clinician provision of support and sharing emotion proven to be more beneficial to patients than sharing information
Edwards et al. [33]	Primary Care	Yes	96	• Including patients in risk estimates during discussion between patients and clinicians regarding genetic screening results is productive
Egan et al. [34]	Alzheimer’s Disease	Not Clear	13	• Employing memory aids and specific caregiver training programs enhances verbal communication, specifically information uptake with patients with Alzheimer’s Disease
Eggenberger, Heimerl & Bennett [35]	Dementia	Yes	12	• Enhancing communication skills of professionals working with dementia patients results in improvements of patient quality of life, positive interactions with peers, and organization of care
Fawole et al. [36]	Palliative Care	Yes	20	• Improving palliative care communication with patients includes improving healthcare utilization and patient/family consultations
Finke, Light & Kitko [37]	Nursing	Not Clear	12	• Improving communication between nurses and non-verbal patients is necessary to reduce patient frustration
Hancock et al. [38]	Palliative Care	Not Clear	51	• Patients’ perceptions of shared information are inconsistent with healthcare professional’s perceptions of the information provided
				• Healthcare professionals “underestimate” patient need for information and “overestimate” patient understanding of illness
Harrington, Noble & Newman [39]	Primary Care	Yes	25	• Improvements in perceptions of autonomy impacts information recall, adherence, attendance, and clinical outcomes following intervention studies aimed to augment patient participation in medical interactions
Henry et al. [40]	Primary Care	Yes	26	• Increased patient satisfaction was correlated with positive/warm clinician interactions with active listening
Janssen & Largo-Janssen [41]	Gynecology	Yes	9	• Patient-centered communication styles increase patient satisfaction
Laidsaar-Powell et al. [42]	Primary Care	Yes	52	• Triadic communication (patient-clinician-family member) involves: encouraging family involvement in care, re-enforcing positive family contributions, identifying roles of patients and family members
Oliveira et al. [43]	Primary Care	Yes	27	• Communication indicating valuing patient autonomy is correlated with high patient satisfaction
Parker et al. [44]	Palliative Care	Not Clear	123	• At end-of-life, patients want less information sharing and caregivers want more information sharing
				• Patients value empathic and honest clinicians who encourage questions and facilitate discussions
Pinto et al. [45]	Rehabilitation	Yes	12	• The “therapeutic alliance” is enhanced by emotional support provision and patient participation during consultation
Rodin et al. [46]	Oncology	Yes	21	• Patients have varying communication needs and may prefer professional-centric communication over patient-centered communication, therefore clinicians are encouraged to individualize their communication styles to patient needs
Scheunemann et al. [47]	Intensive Care	Yes	2841	• Printed communication aids, structured communication from the healthcare team, and ethics consultations improve emotional outcomes for families in the ICU
Slort et al. [48]	Palliative Care	Yes	15	• Clinician availability and openness to facilitating discussions about end-of-life care, including reflection on poor outcomes, facilitates patient-clinician communication
Tay, Hegney & Ang [49]	Nursing	Not Clear	8	• While patient and clinician characteristics are found to influence communication, the role of the environment in effective communication between these two parties is not well documented
				• Reception to patient cues and effective information sharing builds relationships with patients and maintains open communication
Thompson & McCabe [50]	Psychiatry	Not Clear	23	• A strong clinician-patient relationship that involves effective communication is correlated with adherence
				• Clinicians wishing to promote patient-clinician collaboration must attempt to find common ground with patients and share decision making roles
Uitterhoeve et al. [51]	Oncology	Not Clear	7	• No correlation was found between effective communication training and patient distress outcomes
Vasse et al. [52]	Dementia	Not Clear	19	• Improving communication with patients with dementia can improve daily care activities and intervention outcomes; however, has little impact on neuropsychiatric symptoms
Wanyonyi & Themessl-Huber [53]	Primary Care	Yes	6	• Clinicians should allocate time to “discover their patients’ psycho-social characteristics” in order to achieve health promotion

### Analysis

Content analysis of all patient-centered care frameworks and models included in this review revealed that all frameworks and models included approaches to achieving the three essential components of patient-centered care: effective communication, partnership, and health promotion (Table 3).

**Table 3:** Data summary form

	**Communication**		**Partnership**		**Health promotion**		
**Article**	**Sharing information**	**Compassion/empathy/empowerment**	**Sensitivity to needs**	**Relationship building**	**Collaboration**	**Case management**	**Resource use**
Ballweg [7]			**x**			**x**	**x**
Berger [8]							**x**
Bickler [9]			**x**	**x**			**x**
Boltz [10]		**x**		**x**		**x**	**x**
Booth & MacBride [11]		**x**	**x**	**x**			**x**
Briggs [12]		**x**	**x**	**x**			**x**
Browne et al. [13]	**x**	**x**				**x**	**x**
Cox [14]			**x**				
deLusignan et al. [15]						**x**	**x**
DiGoia et al. [16]				**x**			
Enguidanos et al. [17]			**x**				**x**
Ford et al. [18]							
Hantho et al. [19]							
Hatzichristou & Tsimtsiou [20]			**x**		**x**		**x**
Kelleher [21]							
Kibicho & Owczarzak [22]							
McCormack [23]	**x**				**x**		
Rosvik et al. [24]					**x**		
van der Eijk et al. [25]			**x**		**x**		
Total	**17/19**		**11/19**	**14/19**	**15/19**	**15/19**	**9/19**
Incorporates category							
Does not incorporate category	**x**						

### Communication

Three components of communication were commonly discussed in the articles reviewed: a) sharing information, b) compassionate and empowering care provision, and c) sensitivity to patient needs.

### Sharing information

Creation of an effective learning environment was cited as a method for supporting patient-centered care in 89.5% of articles reviewed. Many articles discussed effective communication of healthcare information from the clinician to the patient, but also included approaches to effective patient information uptake by the clinician. Effective information uptake was seen as being an essential step in tailoring information to suit patient needs, vulnerabilities, and capacities [11,12]. Active listening, asking open ended questions, and developing functional goals were strategies cited by review articles to achieve effective information uptake [11,12,15].

### Compassionate and empowering care provision

Providing compassionate and empowering care was cited as a component of achieving effective communication in 53% of articles reviewed. Such care is described as being attentive and altruistic, and was emphasized by several review articles and by the sole randomized control trial included in this review [14,16]. As well, these articles described compassionate and empowering care as contributing to the development of a strong clinician-patient relationship based upon patient feelings of autonomy and trust [14,16].

### Sensitivity to patient needs

Strategies on how to be sensitive to patient needs were primarily discussed in the qualitative research articles included in this review. Such strategies included acknowledging and adapting to unique patient identifiers [19,24,25]. For example, clinicians are urged to observe and reflect on fluctuating levels of patient alertness, patient comfort levels in the presence or absence of family members, and different communication barriers such as hearing loss, in order to facilitate clinical interactions [15,19,22]. Of the articles reviewed, 58% identified that careful observation of unique patient characteristics is necessary to providing care that will lead to optimal patient receptiveness and positive health outcomes.

### Partnership

Two components of partnership development were commonly discussed in the articles reviewed: a) relationship building and b) inter-professional collaboration.

### Relationship building

Relationship building was discussed by all article types included in this review. Of the articles reviewed, 74% identified that building relationships with patients and families contributes to understanding what problems the patient is most concerned with and how their illness or injury has affected their life [15,18,23]. The involvement of patients and families in their care builds trust and encourages mutual problem solving [17].

### Inter-professional collaboration

Engaging in inter-professional collaboration to decentralize health care provision was cited as a method of achieving partnership among healthcare professionals in 79% of the articles reviewed. These articles were primarily review articles that described decentralization as a team-based approach to care provision that contributed to efficient and focused care provision [7-10,13,21].

### Health promotion

Achieving health promotion in a patient-centered context requires reflection on how to best support optimal health and care provision through reflection on the patient’s history. The two components of health promotion that were commonly discussed in the articles reviewed as being effective ways to achieve patient-centered care were: a) effective case management and b) efficient use of resources.

### Effective case management

Effective case management was identified by 79% of articles reviewed as being a necessary component of health promotion. Effective case management involves the evaluation of past successes and failures of care in order to best tailor future health initiatives and reduce risk of adverse health outcomes [26]. This process is facilitated by discussions with patients about previous healthcare experiences in order to develop an understanding of how patients respond to certain types of care, such as care requiring follow-up appointments or self-directed home exercises [17,19,20].

### Efficient use of resources

Appropriate organization of resources around patients was cited by 47% of articles included in this review as a way to achieve health promotion. By using resources that best suit patient needs and values, clinicians can tailor treatment plans to best represent how patients are likely to respond to certain interventions [16].

### Secondary review analysis of communication strategies

The secondary review of systematic review articles on communication strategies in healthcare revealed that the majority of articles (68%) explicitly related communication strategies to patient-centered care. Articles that did not explicitly state this relationship through the use of the terms “patient-centered” or “client-centered” care, implied this relationship by identifying how effective communication between patients and healthcare professionals impacts patient satisfaction and health outcomes. The breadth of disciplines from which this literature was found is consistent with the diverse nature of the literature found on patient-centered care frameworks and models. Exploration of key findings revealed that effective communication strategies surrounding information provision and uptake by the healthcare professional, as well as respect for patient autonomy were the main facilitators of a positive clinical interaction.

## Discussion

This scoping review provides an overview of how patient-centered care is conceptualized in the current literature and suggests that the three components of patient-centered care valued by patients are predominantly featured in patient-centered care models and frameworks across different settings, populations, and applications. These core components were approaches to achieving effective communication, partnership and health promotion. While some of the articles reviewed pertained to specific target populations, the frameworks and models that they described were based on similar components of patient-centered care provision. This suggests that the models can be broadly applied. These components were clearly defined by authors, which made common approaches to communication, partnership, and health promotion easily identifiable during the progression of this scoping review’s analysis.

Epstein et al. [26] identify that while patient-centered care is acknowledged by clinicians as an ideal approach to care provision, “what it is and how to measure it” [26] is not clear to clinicians. They suggest that additional research is needed to strengthen the evidence supporting patient-centered care in healthcare [26]. This scoping review provides a foundation for future research by collating and summarizing the theoretical and empirical evidence regarding effective approaches to achieving patient-centered care provision. There is clearly a need for greater emphasis on empirical testing of the health and system impacts of providing patient-centered care in different contexts since the literature reviewed primarily addressed this topic theoretically, and only one randomized control trial was identified. Despite this finding, the consensus around inclusion of communication, partnership, and health promotion, across frameworks identified through this scoping review provides preliminary support that these key features of patient-centered care should be specifically included and evaluated in future studies or in clinician training.

The use of theoretical foundations is considered important in in complex health care issues, but theory has been operationalized more conceptually than empirically within the literature on patient-centered care, as indicated by the fact that only one randomized control trial was identified. This is consistent with findings of how theory has been applied to knowledge translation within the field of rehabilitation. Colquhoun et al. [27] found theoretical frameworks were more commonly used in a generic way rather than as a specific operational tool for defining interventions, processes, expected outcomes or evaluation strategies. Charting the nature of the evidence with respect to the use of patient-centered care frameworks and models suggests a greater need for empirical studies that test the value of providing patient-centered care versus alternatives in a rehabilitation context. Explicit use of the theory would ideally be integrated throughout training processes, materials that operationalize patient-centered care, evaluative instruments that assess its implementation, and all research that seeks to understand how it affects the process and outcomes of care.

Having found a consensus that communication, partnership and health promotion are key aspects to providing patient-centered care, it is important to have rigorous definitions and clear descriptions of what these processes entail, as well as evidence about how to operationally optimize these elements in different contexts and with different patient populations. This study highlighted a rich body of evidence to inform our understanding of communication. However, health promotion and partnership have a generic meaning that is quite broad, as they have been divided as having specific characteristics within patient-centered care. This may cause confusion for clinicians who believe that they are practicing these components of patient-centered care, as their approach may be consistent with the generic meaning of patient-centered care, but inconsistent with the specific steps and components required to operationalize them in a patient-centered way. Thus, fidelity in patient-centered processes may be lost when the concept is disseminated or scaled-up. Furthermore, the lack of consistency between the meaning of health promotion within patient-centered care and other aspects of healthcare warrants further consideration.

It may be that a more inclusive but specific definition would improve this component of patient centered care. For example, health promotion has been defined by the World Health Organization as “the process of enabling people to increase control over their health and its determinants, and thereby improve their health” [28]. However, within the patient-centered care literature it has been defined as developing healthcare plans based on reflection on patient histories for the purposes of health enhancement, risk reduction, and early detection of illness [5]. There are areas of conceptual consensus across these definitions that suggest they promote a common approach [29-53]. However, the patient-centered care definition implicitly refers to the clinical interaction and goals; whereas, the World Health Organization places greater emphasis on determinants of health. Differences in conceptual framing of health promotion make it difficult to isolate studies that investigate the effect of this component of patient-centered care on outcomes. Conversely, there are a substantial number of systematic reviews that name communication as a key focus (in their title) suggesting that communication strategies can be improved by accessing high quality, empirical evidence. This reflects the importance of communication in most aspects of healthcare, and that it is studied as an important concept even where not framed within a patient-centered care framework. From these studies, we were able to determine that the majority of articles published on effective communication strategies in healthcare have a patient-centered focus and that improved outcomes can be expected when health services are designed to implement such strategies.

## Conclusion

While no unifying patient-centered care framework/model was found, a consensus among frameworks and models of different disciplines suggest that three components of patient-centered care have been consistently recognized as critical to the process. Health promotion, communication and partnership have been considered across multiple areas of clinical practice although rarely through empirical studies. This consensus suggests a broadly applicable framework/model of patient-centered care is feasible and together with appropriate operational definitions might advance future empirical studies addressing whether patient-centered care improves outcomes. Studies that attest to the implementation and empirical evaluation of the outcomes of patient-centered care are needed and should at minimum include and measure the three tenets of patient-centered care: communication, partnership, and health promotion.

## Competing interests

The authors declare that they have no competing interests.

## Authors’ contributions

This study was completed as part of the graduate thesis work of MKC who was the principal investigator in this study. MKC and JCM collaborated to create a study design to answer the research questions posed. MKC conducted the scoping review and JCM acted as the second reviewer. VDBH and ML assisted in the review and feedback process during the final production of this manuscript and also acted as supervisory committee members of MKC. JCM served as MKC’s graduate supervisor. All authors read and approved the final manuscript.

## Pre-publication history

The pre-publication history for this paper can be accessed here:

http://www.biomedcentral.com/1472-6963/14/271/prepub
